# Use of sugammadex in patients with neuromuscular disorders: a systematic review of case reports

**DOI:** 10.1186/s12871-019-0887-3

**Published:** 2019-11-19

**Authors:** Usha Gurunathan, Shakeel Meeran Kunju, Lisa May Lin Stanton

**Affiliations:** 1 Department of Anesthesia and Perfusion Services, The Prince Charles Hospital, Rode Road, Chermside, Queensland 4032 Australia; 2 University of Queensland, Brisbane, Australia

**Keywords:** Sugammadex, Neuromuscular diseases, Rocuronium, Neuromuscular blockade, Reversal

## Abstract

**Background:**

Sugammadex is a modified gamma-cyclodextrin that acts by selectively encapsulating free amino-steroidal neuromuscular relaxants. Several case reports have been published on the use of sugammadex in patients with neuromuscular disorders that include neuromuscular junction diseases, myopathies, neuropathies, and motor neurone disorders. The primary aim of this review is to systematically review the evidence on the use of sugammadex in patients with this heterogeneous group of diseases and provide recommendations for clinical practice.

**Methods:**

A systematic electronic search of Medline, Embase and CINAHL databases was done until June 2019, to identify case reports describing the use of sugammadex in adult surgical patients with neuromuscular disorders.

**Results:**

Of the 578 records identified through database searches, 43 articles were finally included for the systematic review. Of these, 17 reports were on patients with myopathy, 15 reports on myasthenia gravis, 9 reports on motor neuron diseases and 2 reports on neuropathies.

**Conclusions:**

Majority of the articles reviewed report successful use of sugammadex to reverse steroidal muscle relaxants, especially rocuronium, in patients with neuromuscular diseases. However, with sugammadex, unpredictability in response and uncertainty regarding optimum dose still remain issues. Quantitative neuromuscular monitoring to ensure complete reversal and adequate postoperative monitoring is strongly recommended in these patients, despite the use of sugammadex.

## Background

Neuromuscular disorders are a large heterogeneous group of diseases that are usually progressive and produce symptoms at widely differing age ranges with varying degrees of severity [1]. They can be classified into motor neuron diseases, neuropathies, neuromuscular junction disorders or myopathies depending on which section of neuromuscular system is affected [1] (Table 1). Epidemiological studies report an increase in the prevalence of neuromuscular disorders worldwide [2–4]. There have been several publications expressing concerns over the choice of muscle relaxants in patients with neuromuscular disorders presenting for surgery but perhaps, the reversal of the effects of muscle relaxants is a greater concern.

**Table 1:** Classification of the neuromuscular disorders

1. Neuromuscular transmission disorders: Myasthenia Gravis, Lambert-Eaton syndrome.	
2. Myopathies: Muscular dystrophies including myotonias- dystrophic and non-dystrophic myotonias, poly- and dermatomyositis, metabolic and mitochondrial myopathies.	
3. Neuropathies: Inflammatory polyneuropathy (Guillain – Barré syndrome), hereditary and toxic polyneuropathy (Charcot-Marie-Tooth disease, Fredreich’s ataxia), multiple sclerosis	
4. Motor neuron diseases: Amyotrophic lateral sclerosis, spinal muscular atrophy, spinal bulbar muscular atrophy	

Sugammadex (Bridion®, Organon/Schering-Plough USA) a modified γ- cyclodextrin, acts by selectively encapsulating free molecules of amino steroidal neuromuscular relaxants such as vecuronium and rocuronium forming 1:1 inclusion complex in the plasma, thereby creating a concentration gradient resulting in the reduction of the relaxant available at the neuromuscular junction [5–7]. The complex is pharmacologically inert, is not affected by acid-base status or temperature [8] and produces no hemodynamic changes [6]. Thus, sugammadex has been found to have a good safety profile so far, compared with neostigmine [9].

Due to its rapid onset of action, sugammadex has enabled rocuronium to be used in difficult intubation scenarios, where traditionally suxamethonium has been the relaxant of choice [10]. Sugammadex also permits the anesthesiologist to use high dose of rocuronium both for rapid sequence induction and intubation [11] as well as to ensure optimal surgical conditions, by enabling a complete motor recovery and reduced need for postoperative ventilatory support [12]. Moreover, the time taken by sugammadex to adequately reverse moderate to deep block has been found to be shorter than that for neostigmine [10]. Hence, the use of sugammadex is being increasingly reported in patients with neuromuscular disorders. However, synthesis of the evidence from these isolated case reports may provide a more meaningful guidance to the anesthesiologists with their management of such patients and to generate new research hypotheses.

The purpose of the following review is to evaluate the evidence supporting the use of sugammadex as a reversal agent in patients with neuromuscular disorders, in terms of its efficacy and dose requirements and to summarize various aspects that need to be considered during administration of this drug. A detailed review of neuromuscular diseases and their anesthetic considerations is outside the scope of this article.

## Methods

A search was done by the reviewers (U.G and L.S) in Medline, Embase and CINAHL using the key Medical Subject Headings (MeSH) terms, “*sugammadex”, “neuromuscular diseases”, “neuromuscular junction disorders”, “myopathy”, “neuropathy”, “hereditary motor sensory neuropathy”, “motor neuron disease”, “neuromuscular transmission disorders”, “Neuromuscular blocking”* for studies including case reports on adult humans, and published in peer-reviewed journals, without any restriction on the year of publication. The last search was on 24 June 2019. Adult surgical patients with all variants of neuromuscular diseases who received sugammadex for reversal were eligible for inclusion. Paediatric case reports were excluded. Conference abstracts without full text availability and the articles that were not in English were excluded. Controlled trials on sugammadex, studies that did not use neuromuscular monitoring or did not report train-of-four ratio (TOF ratio) or count (TOF count) were excluded. Authors were not contacted for additional information. Duplicates were removed. Full texts of the articles from the relevant abstracts were reviewed. The reference list of the articles thus obtained was manually searched for any additional relevant article by L.S.

**Fig. 1:**
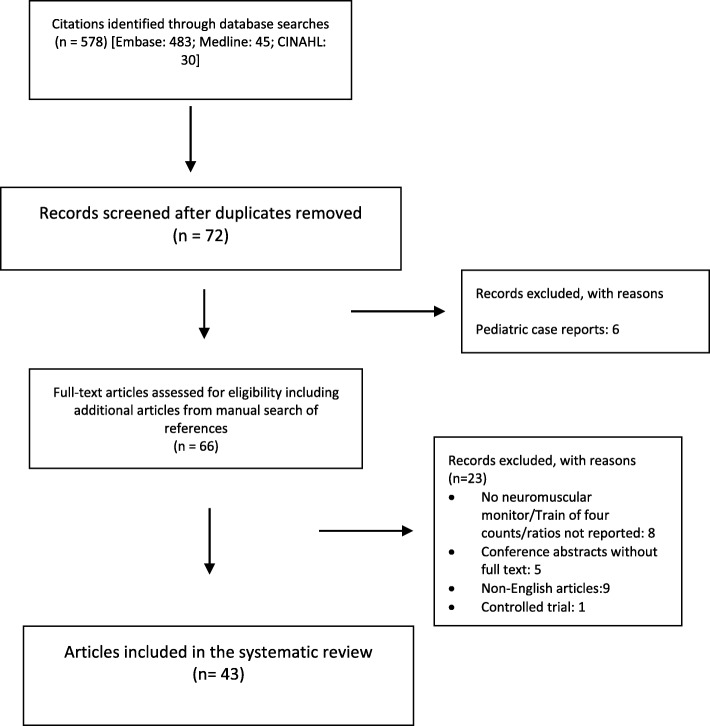
PRISMA flow diagram to illustrate the number of records selected for the systematic review and the reasons for exclusion

Two reviewers (U.G and S.K) independently screened the title and abstracts of all the articles from the literature search to select articles for full-text review with the inclusion and exclusion criteria. Any discrepancy was resolved by mutual consensus and discussion with the reviewer (L.S). Data were extracted by U. G and S. K into an excel sheet and included author, year, country, patient details, nature of disease, type of surgery, duration of surgery, anesthetic agents, neuromuscular blocking agent and its dose, neuromuscular monitor used, dose of sugammadex and its response and postoperative course. Details of the selection process are given in the Preferred Reporting Items for Systematic Reviews and Meta-Analyses (PRISMA) diagram (Fig. 1).

## Results

The search identified 578 citations, 72 relevant abstracts were screened, from which 29 articles excluded, leaving 43 articles suitable for review (Fig. 1). There were 22 publications from Europe, 15 publications from Asia and five from Australia. The maximum number of reports (*n* = 17) concerned patients with myopathies, followed by patients with myasthenia gravis (*n* = 15). One Australian paper [13] reported two cases, of which one concerned a patient with myotonic dystrophy and the other about a patient with spinal muscular atrophy. For the sake of classification, it was considered as two different reports. Two reports were on patients with neuropathies and nine on motor neuron diseases.

## Discussion

Respiratory involvement in neuromuscular disorders can range from a reduction in inspiratory and expiratory muscle strength resulting in alveolar hypoventilation, poor clearance of airway secretions to atelectasis and respiratory failure^1^. There may be coexisting mild to moderate bulbar dysfunction increasing risk of aspiration and obstructive and central sleep apnea [14]. Hence, muscle relaxants have been cautiously or even sparingly used in patients with neuromuscular disorders in order to avoid the need for postoperative ventilatory support. However, inadequate relaxation due to restricted use of muscle relaxants can compromise the success of some abdominal and gynecological procedures [15, 16]. Use of suxamethonium in patients with neuromuscular disorders may risk them with its undesirable side effects such as myalgia, malignant hyperthermia, decreased heart rate, masseter spasm, anaphylaxis, increased intracranial and intraocular pressure, hyperkalemia and prolongation of neuromuscular block in patients with congenital or acquired variations in plasma cholinesterase activity [17, 18]. The response and duration of action of rocuronium can be variable and unpredictable in these patients [19]. Since patients with neuromuscular disorders may also have other associated conditions such as cardiomyopathy [20], systemic and pulmonary hypertension and arrhythmias, the conventional combination of reversal agents (neostigmine and anticholinergic drugs) may cause cardiac rhythm disturbances. Previous case reports have also described prolonged neuromuscular blockade similar to depolarizing block or a tonic response following the use of neostigmine in patients with neuromuscular disorders [21]. Other drawbacks of anticholinesterases such as neostigmine include relatively slow onset along with questionable reliability and predictability of reversal [22].

A recent Cochrane review concluded that sugammadex is faster, more efficient and safer than neostigmine in reversing moderate and deep paralysis [23]^2^. Within our literature search, evidence was collected on the use of sugammadex in four main types of neuromuscular disorders:

### Neuromuscular transmission disorders (Table 2)

Myasthenia gravis is a common autoimmune disorder that can manifest as muscle weakness that is either generalized or isolated to ocular/bulbar muscles. It may also be associated with autonomic instability. Dosing of muscle relaxants may pose challenges in patients with myasthenia gravis. They could be resistant to suxamethonium with up to twice-normal ED_50_ values, with increased risk of phase II blockade at higher doses [24]. In contrast, patients with myasthenia gravis are sensitive to non-depolarizing relaxants due to a decreased number of acetylcholine receptors and hence a dose reduction of these drugs has been recommended [25–27]. In the studies reviewed, the bolus intubating dose of rocuronium used in the patients with myasthenia gravis ranged from 0.09–1.2 mg/kg. Factors such as the use of pyridostigmine and its dose may also impact on the effects and the duration of non-depolarising agents [28]. Moreover, since the acetylcholine esterase is already inhibited by pyridostigmine, reversing residual block with neostigmine may not be fully effective [24, 25, 27]. Use of sugammadex can provide fast and reliable recovery irrespective of preoperative continuation or cessation of pyridostigmine [29]. A large retrospective cohort study has shown a significant reduction in myasthenic crisis and hospital costs following surgery when sugammadex was used [30].

**Table 2:** Summary of case reports on the use of sugammadex in patients with myasthenia gravis (*n* = 15)

Author/year	Country	Patient characteristics	Disease	Type of surgery; Duration of surgery	Anesthetic agents	NMBA	NM monitoring	Dose of sugammadex & results of NM monitor	Postoperative course
de Boer et al., 2010	Netherlands	2 patients-details not provided	Myasthenia gravis	Short procedures -details not provided; N.R.	N.R.	Rocuronium 0.15 mg/kg	Acceleromyography (TOF-Watch SX®)	Sugammadex 4 mg/kg; Pre reversal TOF count: 0 & PTC: 0; Post reversal TOF ratio: 0.9 (2.7 min for the first patient & 2.25 min for the second patient)	Uneventful extubation and recovery
Petrun et al., 2010	Slovenia	44/F; 55 kg, 153 cm; BMI: 23.5 kg/m^2^	Myasthenia gravis	Laparoscopic cholecystectomy; around 30 min	Propofol, sufentanil induction followed by maintenance with sevoflurane/ oxygen/air	Rocuronium 0.36 mg/kg, then 0.18 mg/kg	Acceleromyography (TOF watch S®)	Sugammadex 2 mg/kg; Pre reversal TOF ratio: 0.23; Post reversal TOF ratio: 1(4 min)	Uneventful extubation and recovery
Unterbuchner et al., 2010	Germany	72/M; 88 kg, 172 cm; BMI: 29.7 kg/m^2^	Myasthenia gravis	Elective radical prostatectomy; 210 min	Propofol, sufentanil induction followed by maintenance with propofol infusion and sufentanil bolus	Rocuronium 22 mg initial bolus and another 21 mg before intubation; followed by rocuronium infusion (cumulative rocuronium dose: 151 mg)	Electromyography (NM transmission module in GE Datex Light Monitor)	Sugammadex 2 mg/kg; Pre reversal TOF count:2; Post reversal TOF ratio: 0.9 (3.5 min)	Uneventful extubation and recovery in the intermediate care unit
Argiriadou et al., 2011	Greece	31/F; 95 kg/ 160 cm; BMI: 37 kg/m^2^	Myasthenia gravis	Transsternal thymectomy; 70 min	Propofol, fentanyl induction followed by propofol infusion	Rocuronium 0.5 mg/kg; no further dose	Acceleromyography (TOF-Watch SX®)	Sugammadex 2 mg/kg; Pre reversal TOF ratio: 0.3; Post reversal TOF ratio: 0.92 (3 min), 1.02 (7 min)	Uneventful extubation and recovery
Mitre et al., 2011	Romania	56/F; 90 kg, 179 cm; BMI: 28.1 kg/m^2^	Myasthenia gravis	Laparoscopic cholecystectomy; 40 min	Thiopentone, midazolam and fentanyl induction followed by maintenance with sevoflurane/oxygen/air	Rocuronium 0.6 mg/kg	Acceleromyography (TOF-Watch SX®)	Sugammadex 2 mg/kg; Pre reversal TOF ratio: 0.67; Post reversal TOF ratio: 0.96 (1 min)	Uneventful extubation and recovery
Garcia et al., 2012	France	35/F: 80 kg; 34 weeks gestation	Myasthenia gravis	Emergency cesarean section; 90 min	Propofol, sufentanil induction followed by maintenance with propofol infusion	Rocuronium 8 mg (0.15 mg/kg), modified rapid sequence induction	Qualitative neuromuscular monitoring	Sugammadex 200 mg (4 mg/kg ideal body weight); Pre reversal TOF count: 1; Post reversal TOF count: 4 (4 min)	Artificial ventilation for 48 h due to failure to wean despite good motor response
Jakubiak et al., 2012	Poland	38/F; 160 kg/ 181 cm; BMI: 48.8 kg/m^2^	Myasthenia gravis	Elective laparoscopic adjustable gastric banding; 42 min	Propofol total intravenous infusion	Rocuronium 24 mg (0.15 mg/kg)	Acceleromyography	Sugammadex 200 mg (2 mg/kg corrected body weight) Pre reversal TOF count: 1; Post reversal TOF ratio: 1 (2.8 min)	Uneventful extubation and recovery in both the cases
Üstün et al., 2012	Turkey	2 adult patients: Case 1:55/F; BMI 37 kg/m^2^; Case 2: 45/F; BMI 27 kg/m^2^	Myasthenia gravis	Case 1: Disc Hernia repair; 135 min Case 2: Abdominal hysterectomy; 96 min	Propofol, remifentanil induction followed by maintenance with remifentanil infusion and sevoflurane/oxygen/air	Case1: Rocuronium 0.2 mg/kg for intubation followed by 1/8th of the dose as top up Case 2: Rocuronium 0.25 mg/kg	Acceleromyography (TOF-Watch SX®)	Case 1: Sugammadex 2 mg/kg; Pre reversal TOF: 0.15; Post reversal TOF ratio: 1 (2 min). Case 2: Sugammadex 2 mg/kg; Pre reversal TOF count: 2; Post reversal TOF ratio: 1 (5 min)	Uneventful extubation and recovery
Iwasaki et al., 2013	Japan	2 patients. Case 1: 74/F; 54 kg/157 cm; BMI: 21.9 kg/m^2^. Case 2: 71/M, 72 kg/165 cm; BMI: 26.4 kg/m2	Ocular myasthenia gravis	Case 1: Capsulosynovectomy left elbow; N.R. Case 2: Transcervical thymectomy; N.R.	Case 1: Propofol induction followed by maintenance with propofol and remifentanil Case 2: Propofol induction followed by maintenance with sevoflurane, remifentanil along with epidural anaesthesia (T5-T6 level)	Case 1: Rocuronium 0.5 mg/kg; additional 0.2 mg/kg if TOF count ≥2 Case 2: Rocuronium 0.3 mg/kg; additional 0.15 mg/kg if TOF count ≥2	Acceleromyography (TOF-Watch SX®)	Case 1: Sugammadex 2 mg/kg followed by two additional boluses of 1 mg/kg; Pre reversal TOF ratio:0.2; Post reversal TOF 0.9 (1.5 min)^a^. Subsequently two additional boluses of 1 mg/kg sugammadex were administered Case 2: Sugammadex 1 mg/kg followed by two additional boluses of 1 mg/kg; Pre reversal TOF count: 2; Post reversal TOF ratio 0.9 (6.5 min)^a^ (after 2 mg/kg sugammadex)	Uneventful extubation and recovery
Kiss et al., 2013	Switzerland	25/F; BMI: 32.0 kg/m2	Myasthenia gravis	Thymectomy; 120 min	Propofol infusion and sufentanil	Rocuronium 30 mg for intubation along with two 10 mg boluses (total 50 mg)	Datex Ohmeda M-NMT module and portable neuromuscular stimulator	Total dose of sugammadex: 17.34 mg/kg; Pre reversal TOF ratio: 0.36, Post reversal TOF ratio: 0.71 (after more than 8 min).	Pyridostigmine was given through nasogastric tube. Extubation after long waiting time, at the end of surgery
Sugi et al., 2013	Japan	26 yr/F; 64 kg; 165 cm	Myasthenia gravis	Extended thymectomy; 155 min	Induction and maintenance with TCI propofol and remifentanil infusion supplemented with fentanyl boluses	Rocuronium 6 mg (0.09 mg/kg) for intubation; Total dose of rocuronium 28 mg.	Acceleromyography (TOF-Watch SX®)	Sugammadex 2 mg/kg. Post reversal TOF ratio: 0.55 (8.5 min). Additional 2 mg/kg sugammadex administered: No change in TOF ratio. Further supplemented with neostigmine 0.3 mg/kg; Post neostigmine TOF ratio: 0.86 (5 min). Post reversal TOF ratio (3 h & 7 h): 0.8 & 0.9 respectively.	Extubated after a delay at the end of surgery; Uneventful recovery
Sungur Ulke et al., 2013	Turkey	10 patients: mean age: 31 ± 12 years; Weight: 68 ± 13 kg	Myasthenia gravis	Video thoracoscopic assisted thymectomy; mean surgical time: 62 +/− 16 min	Propofol, Fentanyl induction followed by propofol infusion & fentanyl boluses	Rocuronium 0.3 mg/kg; Mean total dose of rocuronium: 48+/− 16 mg	Acceleromyography (TOF-Watch S®)	Sugammadex 2 mg/kg; Pre reversal TOF ratio: ranged from 0 to 0.5; Mean time to TOF > 0.9: 1.85 min.	Uneventful extubation and recovery
Casarotti et al., 2014	Italy	2 patients: Case 1: 48/M; BMI: 32.7 kg/m^2^. Case 2: 71/F	Myasthenia gravis	Case 1: Emergency laparotomy; 120 min Case 2: Emergency endoscopy for hemostasis; 60 min	Propofol, remifentanil induction followed by maintenance with propofol and remifentanil infusion	Case 1: Rocuronium 1.2 mg/kg ideal body weight. Rapid sequence induction Case 2: Rocuronium 1 mg/kg ideal body weight Rapid sequence induction	Acceleromyography (TOF-Watch SX®)	Case 1: Sugammadex 4 mg/kg actual body weight. Pre reversal PTC > 1. Post reversal TOF ratio: 0.9 (3 min) Case 2: Sugammadex 4 mg/kg actual body weight. Pre reversal TOF count: 1; Post reversal TOF ratio: 1 (2 min)	Intensive care unit monitoring; sedated for at least 30 min after sugammadex and then extubated; uneventful recovery.
de Boer et al., 2014	Netherlands, UK	21 patients; M: 8; F: 13. Mean age: 56 years Average weight: 77.6 kg	Myasthenia gravis	Thymectomy: 10 Breast surgery: 3; Laparoscopic cholecystectomy: 1; Urological surgery: 2, Craniotomy: 1; Laminectomy: 1; Inguinal hernia repair: 1; Gastric surgery: 1; Skin lesions: 1; Duration: N.R.	Propofol, remifentanil induction and maintenance or propofol induction and sevoflurane for maintenance	Rocuronium: 13 patients: 0.1–1.0 mg/kg; Vecuronium: 8 patients: 0.1–0.2 mg/kg	Acceleromyography (TOF-Watch SX®)	a) Sugammadex 2 mg/kg for 12 patients. Pre reversal TOF count: ≥1 response; Post reversal TOF ratio: 0.9 (1.3 min) b) Sugammadex 4 mg/kg for 9 patients. Pre reversal TOF count: 0. Post reversal TOF ratio: 0.9 (2.75 min)	Uneventful extubation and recovery in all patients.
Vymazal et al., 2015	Czech Republic	117 patients; M: 67, F: 50; Mean age: 41.6 years; Mean BMI: 24.2 kg/m2	Myasthenia gravis	105 patients: Surgical thymectomy, 12 patients: cholecystectomy; mean surgical time: 98.6 min	Propofol, sufentanil boluses; isoflurane/oxygen/air	Rocuronium 0.6 mg/kg for intubation with additional boluses of 0.15 mg/kg if required; Total dose of rocuronium: 72.5 mg.	Acceleromyography (TOF-Watch SX®)	Sugammadex 2 mg/kg (if pre reversal TOF count: ≥2) or 4 mg/kg (if pre reversal TOF count: 0–1); Post reversal TOF ratio: 0.9 (average 1.95 min)	Uneventful extubation and recovery

*TOF* Train of four, *PTC* Post tetanic count, *N.R.* Not reported, *NMBA* Neuromuscular blocking agent, *NM* Neuromuscular

^a^Results at the adductor pollicis muscle

In the literature reviewed, the documented dosing of sugammadex was also found to vary between reports (Table 2). A dose of 2 mg/kg sugammadex has been noted to be sufficient even with a TOF count of 0 at the time of administration [31] whereas a dose of 4 mg/kg has been used by other authors [29, 32]. In the largest case series to date on the use of sugammadex in myasthenic patients, administration of sugammadex at 2 or 4 mg/kg depending on a TOF count to ≥2 or 0–1 respectively, resulted in full reversal with a duration of less than 2 min on average [33].^3^ However, as per the other reports in our review, complete reversal of relaxant effect occurred within around 3–4 min following sugammadex administration. Interestingly, four reports [32, 34–36] describe persistent residual paralysis in patients with myasthenia gravis even after administration of sugammadex. Kiss et al. [34] described the persistence of neuromuscular blockade in a patient with myasthenia gravis, resulting in the administration of a total dose more than 16 mg/kg, in addition to administration of pyridostigmine via nasogastric tube. This was attributed to both redistribution of muscle relaxant and artifact from neuromuscular monitors. Surgery-induced exacerbation of myasthenia gravis has also been noted to result in residual paralysis despite a sugammadex dose of 4 mg/kg [32, 35].

In terms of monitoring the adequacy of reversal, motor recovery can occur later at the corrugator supercilii muscle (CSM) than at the adductor pollicis muscle (APM) in patients with ocular myasthenia gravis as opposed to individuals without the disease [37].^4^ In addition, recovery of TOF ratio may be faster than that of first twitch (T1) height after sugammadex administration as observed by Iwasaki et al. in two patients with myasthenia gravis.^4^ While the TOF ratio at the APM returned to 90% within 1.5 min and 6.5 min in their two patients, T1 recovery took up to 12 min and 13 min respectively and required additional doses of sugammadex.^4^ Hence the authors recommended monitoring TOF ratio as well as the recovery of T1 height to baseline at both APM and CSM, in patients with myasthenia gravis [37]. However, recovery of TOF ratio was found to lag behind T1 recovery in the case reported by Sugi et al. [35].^4^

### Myopathies (Table 3)

Muscular dystrophies are a heterogenous group of progressive neuromuscular disorders resulting from genetic mutations that cause dystrophic changes in muscles. The most common varieties are Duchenne, Becker and myotonic dystrophies [38]. Patients suffer varying patterns of skeletal muscle weakness depending on the mutation, cardiac abnormalities including cardiomyopathies with or without conduction defects and are prone to pulmonary infection and failure. Myotonic dystrophy is also characterized by prolonged contraction of muscle with defective relaxation. Renal dysfunction may be a common complication in patients with myotonic dystrophy [39]. Patients with myotonic dystrophy tend to show myotonic responses to suxamethonium [40] and increased sensitivity to non-depolarising muscle relaxants [41]. Reactions to neostigmine can also be unpredictable [21, 41]. None of these reactions were observed by Imison et al. in the retrospective study on myotonic dystrophy patients [42].

**Table 3:** Summary of case reports on the use of sugammadex in patients with myopathies (*n* = 17)

Author/year	Country	Patient characteristics	Disease	Type of surgery; Duration of surgery	Anesthetic agents	NMBA	NM monitoring	Dose of sugammadex & results of NM monitor	Postoperative course
Baumgartner, 2010	Australia	59/M; 75 kg	Classic severe myotonic dystrophy	Elective laparoscopy; 46 min	Propofol, alfentanil for induction followed by maintenance with fentanyl boluses sevoflurane	30 mg (0.4 mg/kg) rocuronium given after intubation	Qualitative neuromuscular monitoring	Sugammadex 150 mg; Pre reversal TOF count: 0 with myotonic response to tetany; Post reversal TOF count: 4 equal twitches (4 min)	Extubated end of surgery (within 10 min of sugammadex dose)
Matsuki, Y et al., 2011	Japan	24/F; 75 kg; 160 cm.	Myotonic dystrophy	Laparoscopic ovarian cystectomy; N.R.	Propofol, remifentanil induction followed by maintenance with propofol, remifentanil infusion	Rocuronium 0.3 mg/kg followed by 0.1 mg/kg with the appearance of 4th twitch	Acceleromyography (TOF-Watch SX®)	Sugammadex 2 mg/kg; Pre reversal TOF count: 2; Post reversal TOF: 0.9 (< 2 min)	Extubation at the end of surgery
Mavridou et al., 2011	Greece	40/F; 74 kg; 160 cm; BMI: 28.9 kg/m^2^	Myotonic dystrophy	Laparoscopic cholecystectomy and right ovarian cystectomy; 90 min	Propofol induction followed by propofol, remifentanil infusion with oxygen/air	Rocuronium 30 mg (0.4 mg/kg)	Acceleromyography (TOF-Watch SX®)	Sugammadex 2 mg/kg; Pre reversal TOF count: 2; Post reversal TOF ratio: 1.0 (2 min)	Mechanically ventilated for around 25 min due to pethidine induced respiratory depression, which was reversed with naloxone; Uneventful extubation; No complications thereafter.
Petrovski, 2011	Australia	43/F; BMI: 55 kg/m2	Myotonic dystrophy	First surgery: Cystoscopy & colonoscopy; 90 min Second surgery: urological procedure; 180 min	First surgery: Propofol and sevoflurane/oxygen induction followed by maintenance with desflurane/oxygen/air with fentanyl. Second surgery: Details not reported, other than 200 mcg fentanyl	First surgery: Rocuronium 50 mg for intubation; Second surgery: Rocuronium 50 mg + Cisatracurium 4 mg	Qualitative neuromuscular monitoring	First surgery: Pre reversal TOF count 4; Sugammadex 200 mg; Post reversal TOF: N.R. Second surgery: Pre reversal TOF count:4; Reversal with Neostigmine 2.5 mg & Glycopyrollate 0.4 mg. Post reversal TOF: strong 4 twitches, however clinical signs of inadequate muscle strength recovery	First surgery: Uneventful extubation; Second surgery: Failed extubation, requiring 3 h of ventilation and postoperative lung infection.
Suzuki et al., 2012	Japan	75 yr/M	Dermatomyositis	Open reduction of fracture elbow; 25 min	Propofol, fentanyl induction followed by maintenance with sevoflurane, remifentanil infusion and fentanyl boluses	Rocuronium 0.6 mg/kg	Acceleromyography (TOF-Watch SX®)	Sugammadex 2 mg/kg; Pre reversal TOF count: 1; Post reversal TOF: 0.9 (5.75 min).	Uneventful extubation and recovery
Kashiwai et al., 2012	Japan	37/F; 55 kg; 154 cm	Myotonic dystrophy	Open resection of ovarian tumor	General anesthesia with fentanyl and propofol target- controlled infusion followed by maintenance with propofol, remifentanil infusions and intermittent epidural ropivacaine	Rocuronium 1 mg/kg followed by a subsequent bolus of 0.2 mg/kg	Acceleromyography (TOF-Watch SX®)	Sugammadex 2 mg/kg; Pre reversal TOF count: 2; Post reversal TOF: 0.9 (1.5 min)	Uneventful extubation and recovery
Carron et al., 2013	Italy	67/F; 60 kg, 155 cm; BMI: 25 kg/m2	Polymyosits with Sjogren’s syndrome	Laparoscopic sigmoid resection for diverticulitis; 210 min	Propofol, fentanyl induction followed by maintenance with desflurane and remifentanil	Rocuronium 0.9 mg/kg bolus followed by additional boluses to a total dose of 220 mg	Acceleromyography (TOF-Watch SX®)	Sugammadex 4 mg/kg; Pre reversal TOF count: 0, PTC: 1; Post reversal TOF ratio: 1.1(1.5 min)	Uneventful extubation and recovery
*Stewart et al., 2013	Australia	38/F; 76 kg; 165 cm; BMI: 27.9 kg/m^2^	Myotonic dystrophy	Laparoscopic cholecystectomy; 65 min	Propofol, remifentanil induction followed by maintenance with propofol and remifentanil infusion, oxygen / air	Rocuronium 35 mg (0.47 mg/kg); Rapid sequence induction with cricoid pressure	Kinemyography TOF monitor (M-NMT, Datex Ohmeda, Finland)	Sugammadex 200 mg (2.7 mg/kg); Pre reversal TOF count: 2; Post reversal TOF: 0.9 (5 min)	Uneventful extubation and recovery; Post-operative monitoirng in intensive care unit
Stourac et al., 2013	Czech Republic	? 32/F; 38 weeks gestation	Myotonic dystrophy	Elective cesarean section; 55 min	Propofol induction followed by maintenance with sevoflurane	Rocuronium 1 mg/kg	Acceleromyography (TOF-Watch SX®)	Sugammadex 4 mg/kg; Pre reversal TOF count: 0; Post reversal TOF ratio: 0.9 (2 min)	Uneventful extubation and recovery, both mother and child
Wefki Abdelgawwad Shousha et al., 2014	Italy	25/M; BMI: 25.6 kg/m^2^	Duchenne Muscular dystrophy	Open cholecystecomy; 240 min	Propofol, fentanyl induction followed by maintenance with fentanyl, sevoflurane/oxygen/air	Rocuronium 10 mg to facilitate rapid sequence intubation followed by 5 mg every 45 min.	Acceleromyography (TOF Guard)	Sugammadex 150 mg; Pre reversal TOF ratio: 0.25; Post reversal TOF ratio: 0.9 (10 min)	Uneventful extubation and recovery
Shimauchi et al., 2014	Taiwan	54/M; 54 kg, 167 cm; BMI: 19.4 kg/m^2^	Becker’s muscular dystrophy	Laparoscopic cholecystectomy; 92 min	Fentanyl, midazolam induction; maintenance with propofol, remifentanil infusion, oxygen/air	Rocuronium 20 mg (0.4 mg/kg) followed by bolus to a total dose of 30 mg	Acceleromyography (TOF-Watch SX®)	Sugammadex 100 mg (2 mg/kg); Pre reversal TOF ratio: 0.2; Post reversal TOF ratio: 1.0 (2 min)	Uneventful extubation and recovery
Gurunathan & Duncan, 2015	Australia	60/M; 70 kg	Myotonic dystrophy	Laparoscopic cholecystectomy; 45 min	Propofol, midazolam, Remifentanil infusion for induction followed by maintenance with propofol and remifentanil infusion	Rocuronium 50 mg	Qualitative neuromuscular monitoring	Sugammadex 200 mg (approx 3 mg/kg); Pre reversal TOF count: 0; Post reversal TOF count: 4 twitches (0.5 min)	Uneventful extubation and recovery
Kendigelen et al., 2015	Turkey	52/M; 75 kg	Dermatomyositis	Ileostomy; 110 min	Propofol, remifentanil induction followed by maintenance with remifentanil infusion along with sevoflurane/oxygen/air	Rocuronium 0.6 mg/kg followed by 10 mg bolus	Acceleromyography (TOF-Watch SX®)	Sugammadex 2 mg/kg (150 mg); Pre reversal TOF ratio: 0.40; Post reversal TOF ratio: 0.9 (1 min)	Uneventful extubation and recovery
Kosinova et al., 2016	Czech Republic	27/F; 90 kg; 39^+ 4^ weeks gestation	Becker’s myotonia congenita	Elective caesarean section; around 40 min	Propofol target controlled infusion, sufentanil	Rocuronium 1 mg/kg	Acceleromyography (TOF-Watch SX®)	Sugammadex 4 mg/kg; Pre reversal TOF: 0; Post reversal TOF: 0.98 (2 min 15 s)	Uneventful extubation and recovery
Creaney et al., 2018	Ireland	25/F; 61 kg; 146 cm; BMI: 28.6 kg/m^2^; 30^+ 6^ weeks gestation	Congenital muscular dystrophy	Elective caesarean section; N.R.	Intravenous dexmedetomidine slow bolus followed by maintenance infusion throughout the procedure. Propofol 180 mg induction followed by a maintenance of propofol target controlled infusion; humidified high flow nasal oxygen	Rocuronium 1 mg/kg	Qualitative neuromuscular monitoring	Sugammadex 12 mg/kg in total; Pre reversal TOF count: 0; Post reversal TOF count: 4 (5 min)	Transferred to intensive care unit with dexmedetomidine infusion and extubated to non-invasive ventilation later, with pre-pregnancy BiPAP settings achieved in 24 h.
Teixeira et al., 2019	Portugal	37/M; 65 kg; 173 cm	Myotonic dystrophy type 1 (Steinert disease)	Laparoscopic cholecystectomy; 60 min	Propofol and remifentanil target-controlled infusion for induction and maintenance	Rocuronium 25 mg (0.04 mg/kg)	Acceleromyography	Sugammadex: 150 mg (appr 2.3 mg/kg); Pre reversal TOF count: 2; Post reversal TOF count: 4, ratio: 0.96 (< 5 min)	Uneventful extubation and recovery
Mangla et al., 2019	USA	46/F; 63 kg; 170 cm	Myotonic dystrophy	Robotic assisted laparascopic total abdominal hysterectomy and bilateral salpingo-oophorectomy; 3 h	Propofol induction followed by maintenance with fentanyl bolus, propofol and remifentanil infusions	Rocuronium 30 mg (0.48 mg/kg)	Qualitative neuromuscular monitoring (orbicularis oculi muscle)	Sugammadex 240 mg (3.8 mg/kg); Pre reversal TOF count: 0 (only weak post-tetanic counts were present); Post reversal TOF count:? 4 (not clearly stated) (10 min)	Uneventful extubation and recovery

*TOF* Train of four; *PTC* Post tetanic count; *N.R* Not reported; *NMBA* Neuromuscular blocking agent; *NM* Neuromuscular

Ten reports discussed the use of sugammadex patients with myotonic dystrophy and two reports in patients with Becker and Duchenne muscular dystrophy [20, 43] (Table 3). The dose of rocuronium have been very variable with these studies. Reduced doses (< 0.6 mg/kg) of rocuronium have been administered to aid intubation in majority of the cases in our review ^3^ [13, 20, 43–49]. With these cases, the reversal times to TOF ratio of 0.9 with 2 mg/kg sugammadex ranged from 2 min [20, 45, 46] to 5 min [13, 44, 48]. However, two authors have reported delayed neuromuscular recovery time of 10 min [43, 49], but no explanations were given. It is possible that slight underdosing of sugammadex could have contributed to the delay with Mangla et al. [49]. Three authors had used 1 mg/kg rocuronium to aid intubation, possibly related to their rapid sequence induction [50–52]. With all these cases, standard recommended doses of sugammadex were administered according to the TOF count and TOF ratio of 0.9 was reached within reasonable time (< 3 min).^3^ While response times to both rocuronium and sugammadex were not delayed, Stourac et al. observed prolonged duration of paralysis with rocuronium [51].

Polymyositis and dermatomyositis cause symmetrical weakness of proximal muscles due to an inflammatory process of the muscle itself with no impact on neuromuscular junction. However, two case reports on the use of sugammadex in patients with dermatomyositis, describe a delay in complete neuromuscular blockade (up to around 5 min), which the authors attribute to vascular pathology associated with the disease process resulting in slow diffusion of rocuronium to neuromuscular junction [53, 54] (Table 3). Prolonged reversal time with sugammadex [54] was observed by Suzuki et al. while the other two reports concluded that reversal time was unaffected in these inflammatory myopathies.

### Neuropathies (Table 4)

A number of neuromuscular disorders could be grouped under neuropathies. One report on transverse myelitis and one report on multiple sclerosis were selected for this review (Table 4). Multiple sclerosis is a frequently occurring demyelinating neuropathy. The reports on multiple sclerosis patients did not suggest an altered dose of rocuronium or unusual response to sugammadex. However, a resistance to rocuronium was described by Staikou et al. manifesting as delay in onset of action following 1 mg/kg of rocuronium [55]. Transverse myelitis involves myelin destruction due to spinal cord inflammation. Prolonged paralysis was reported in a patient with transverse myelitis following the administration of 1.2 mg/kg rocuronium for rapid sequence induction [56].

**Table 4:** Summary of case reports on the use of sugammadex in patients with neuropathies (*n* = 2)

Author/year	Country	Patient characteristics	Disease	Type of surgery; Duration of surgery	Anesthetic agents	NMBA	NM monitoring	Dose of sugammadex & results of NM monitor	Postoperative course
Weekes et al., 2010	Ireland	38/F; 70 kg	Idiopathic transverse myelitis	Elective cesarean section; 60 min	Thiopentone and rapid sequence induction followed by maintenance with morphine, sevoflurane/oxygen/nitrous oxide; propofol infusion during delayed extubation	Rocuronium 1.2 mg/kg	Qualitative neuromuscular monitoring	Initial neostigmine 5 mg (0.07 mg/kg) & glycopyrollate 1 mg; Pre reversal TOF: four weak TOF twitches^a^; Post reversal TOF: 4 weak twitches (for more than 1 h). Sugammadex 4 mg/kg administered (delayed administration because of unavailability) followed by all the clinical signs of adequate recovery in 2 min	Uneventful extubation and recovery
Staikou and Rekatsina, 2017	Greece	31/F; 62 kg; 164 cm; BMI: 23.1 kg/m^2^	Multiple sclerosis	Myomectomy; 65 min	Benzodiazepine premedication. Propofol, fentanyl induction followed by maintenance with fentanyl boluses, sevoflurane, nitrous oxide/oxygen	Rocuronium 1 mg/kg for intubation with no further doses	Neuromuscular module of S/5 anaesthesia monitor	Sugammadex 2 mg/kg; Pre reversal TOF count: 3; Post reversal TOF ratio: 0.9 (0.75 min)	Uneventful extubation and recovery.

*TOF* Train of four; *PTC* Post tetanic count; *N.R.* Not reported; *NMBA* Neuromuscular blocking agent; *NM* Neuromuscular

^*a*^*Using facial nerve*

### Motor neuron diseases (Table 5)

Motor neuron diseases are a group of disorders characterized by progressive motor neuron degeneration, the most common of which is amyotrophic lateral sclerosis (ALS). It mainly involves lower motor neurons although in ALS both upper and lower motor neurons are affected [57]. In a patient with ALS reported by Kelsaka et al., clinical signs of inadequate recovery were observed despite a TOF ratio > 0.90. Two minutes after sugammadex 2 mg/kg was administered, the patient recovered clinically and was extubated uneventfully [58] (Table 5). A similar discrepancy between TOF ratio and clinical signs of muscle strength recovery was also reported by Chang et al. [59, 60].^5^ These authors henceforth questioned the reliability of TOF ratio to guide extubation in patients with this condition and proposed that this discordance may be related to the site and the severity of disease [59–61]. In fact, in patients reversed with sugammadex, a TOF ratio of 0.9 may not guarantee complete reversal without complete recovery of first twitch height (T1) [62]. Interestingly, no such issue was noticed by Yoo et al. in their patients with ALS or progressive muscle atrophy (PMA) in spite of their preexisting bulbar dysfunction. However, they had administered 5 mg/kg sugammadex as the pre-reversal TOF count was zero [63].

**Table 5:** Summary of case reports on the use of sugammadex in patients with motor neuron diseases (*n* = 9)

Author/year	Country	Patient characteristics	Disease	Type of surgery; Duration of surgery	Anesthetic agents	NMBA	NM monitoring	Dose of sugammadex & results of NM monitor	Postoperative course
Vilela et al., 2012	Portugal	61/M; 85 kg, 175 cm; BMI: 27.8 kg/m^2^	Spinal muscular atrophy	Elective percutaneous atrial septal defect (ostium secundum) closure; 117 min	Propofol, remifentanil induction followed by maintenance with propofol, remifentanil infusion	Rocuronium 40 mg (0.47 mg/kg)	Acceleromyography (TOF-Watch SX®)	Sugammadex 170 mg (2 mg/kg); Pre reversal TOF ratio: 0.62, Post reversal TOF ratio: 0.90 (69 s)	Uneventful extubation and recovery
Franco-Hernández et al., 2013	Spain	2 siblings; Case 1: 47/F Case 2: 43/F	Strumpell-Lorrain Disease/Familial spastic paraplegia	Case 1: Cholecystectomy; N.R. Case 2: Laparoscopic subtotal colectoy and ileostomy; N.R.	Propofol, midazolam, Fentanyl induction (both) followed by maintenance with sevoflurane, remifentanil infusion (Case 1) propofol and remifentanil infusion (Case 2)	Rocuronium 0.6 mg/kg; no further boluses	Quantitative neuromuscular monitoring	Sugammadex 2 mg/kg; Pre reversal: moderate neuromuscular blockade; Post reversal TOF ratio: > 0.9	Uneventful extubation and recovery in both cases
Kelsaka et al., 2013	Turkey	47/M; 70 kg	Amyotrophic lateral sclerosis (Lou Gehrig’s disease)	Fracture neck of humerus; 75 min	Propofol, remifentanil induction followed by maintenance with remifentanil infusion, sevoflurane/oxygen/air	Rocuronium 20 mg (0.29 mg/kg) for intubation; Additional 10 mg bolus during the procedure	Acceleromyography (TOF-Watch SX®)	Sugammadex 2 mg/kg; Pre reversal TOF > 0.9 with spontaneous breathing but difficulty in opening eyes; Post reversal TOF not stated; but increase in depth of breathing and able to open eyes spontaneously after 2 min.	Uneventful extubation and postoperative monitoring in intensive care unit
^a^Stewart et al., 2013	Australia	61/F; 40 kg; 162 cm; BMI: 15.2 kg/m^2^	Spinal muscular atrophy	Combined approach tympanoplasty; 118 min	Propofol, remifentanil induction followed by maintenance with propofol and remifentanil, oxygen / air	Rocuronium 40 mg/kg (1 mg/kg); rapid sequence induction	Acceleromyography (TOF-Watch SX®)	Reversal was administered after 17 min to assist surgery. Sugammadex 160 mg (4 mg/kg); Pre reversal TOF ratio: 0, post-tetanic count 1; Post reversal TOF: 0.9 (2.8 min).	Uneventful extubation and recovery
Takeuchi, R et al., 2014	Japan	62/M; 70 kg, 173 cm; BMI: 23.4 kg/m^2^	Kennedy’s disease (Spinal bulbar muscular atrophy)	Frontal sinusectomy; N.R.	Propofol, remifentanil induction followed by maintenance with propofol and remifentanil infusion, oxygen / air and fentanyl bolus end of surgery	Rocuronium 40 mg (0.57 mg/kg)	Qualitative neuromuscular monitoring	Sugammadex 150 mg (2 mg/kg); Pre reversal TOF count: 1; Post reversal TOF count: 4 (3 min)	Extubation 5 min after sugammadex; Uneventful recovery
Chang et al., 2014	Korea	47.M; 38 kg; 165 cm; BMI: 14 kg/m^2^	Amyotrophic lateral sclerosis	Total thyroidectomy with cervical node dissection; anaesthesia time 405 min	Propofol, remifentanil target-controlled infusion for induction and maintenance oxygen / air and fentanyl bolus end of surgery	Rocuronium 0.3 mg/kg for intubation with subsequent boluses of 10 and 5 mg	Acceleromyography (TOF-Watch SX®)	Sugammadex 1 mg/kg; Pre reversal TOF: 0.98, but with inadequate tidal volume and difficulty in opening eyes spontaneously. Post sugammadex, adequate clinical signs of recovery from paralysis.	Uneventful extuation; Postoperative ICU monitoring for 4 days
Chang et al., 2017	Korea	62/F; 52 kg; 167 cm; BMI: 18.6 kg/m^2^	Amyotrophic lateral sclerosis	Ureteroscopic ureterolithotomy; 84 min	Propofol induction followed by maintenance with sevoflurane, oxygen/air. No details on opioids	Rocuronium 20 mg bolus (0.38 mg/kg)	Acceleromyography (TOF-Watch SX®)	Sugammadex 100 mg (1.92 mg/kg); Pre reversal TOF: 0.65; Post reversal TOF: > 0.90 (80 s). In spite of TOF > 0.9, additional 100 mg (1.92 mg/kg) sugammadex administered due to reduced tidal volume and muscle strength with no improvement.	Postoperative transfer to ICU and ventilated for 4 hours followed by uneventful extubation.
Yoo et al., 2017	Korea	Case 1: 54/M; 70 kg; 175 cm; BMI: 23 kg/m^2^ Case 2: 66/F; 40 kg; 154 cm; BMI: 17 kg/m^2^	Case 1: Progressive muscular atrophy Case 2: Amyotrophic lateral sclerosis	Case 1: Removal of intramedullary nail left femur and plate left humerus; 160 min Case 2: Split thickness skin grafting lower limb; 60 min	Case 1 &2: Premedication with glycopyrollate. Propofol with lignocaine induction, continuous remifentanil infusion; maintenance with desflurane and fentanyl bolus at the end of surgery.	Case 1: Rocuronium 30 mg (0.43 mg/kg) for intubation and a subsequent 5 mg bolus. Case 2: Rocuronium 20 mg (0.5 mg/kg) for intubation and a subsequent 5 mg bolus.	Quantitative neuromuscular monitoring	Case 1: Sugammadex 200 mg (2.86 mg/kg). Pre reversal TOF: 0.15 Post reversal TOF 1.25 (3 min) Case 2: Sugammadex 200 mg (5 mg/kg). Pre reversal TOF: 0 Post reversal TOF 1.15 (4 min)	Case 1 and 2: Uneventful extubation and recovery
Tada et al., 2019	Japan	54/F; 48 kg; 156 cm; BMI: 19.7 kg/m^2^	Hereditary spastic paraplegia	Decompressive laminectomy; Duration of surgery: N.R.	Propofol, remifentanil for induction followed by maintenance with fentanyl boluses and remifentanil infusion with desflurane/oxygen/air	Rocuronium 20 mg for intubation followed by 20 mg rocuronium as boluses to a total of 40 mg	TOF -Watch (NIHON KOHDEN Corporation, Japan)	Sugammadex 100 mg (2 mg/kg); Pre reversal TOF: N.R. Post reversal TOF count: 4 (ratio > 0.9)	Uneventful extubation and recovery

^*a*^ *Two cases reported in this paper are given under two different sections*

*TOF* Train of four; *PTC* Post tetanic count; *N.R*. Not reported; *NMBA* Neuromuscular blocking agent; *NM* Neuromuscular

Use of sugammadex has also been investigated in patients with other motor neuron diseases (Table 5). Patients with spinobulbar muscular atrophy (Kennedy’s disease) are at increased risk of laryngospasm and bulbar dysfunction and therefore aspiration [64]. Administration of sugammadex 2 mg/kg with TOF count of 1 has been reported to have resulted in 100% reversal within 180 s in a patient with Kennedy’s disease [65]. Two papers reported the management of patients with spinal muscular atrophy [13, 66]. Although an immediate and adequate response to sugammadex was observed in both these patients, an increased sensitivity and prolonged paralysis from rocuronium was reported by Vilela et al. [66].

Based on our literature search, the implications for the use of sugammadex can be found as endnotes.

### Other relevant considerations of sugammadex

Use of sugammadex does not guarantee adequate recovery unless confirmed by TOFr of at least 0.9^6^. Since sugammadex does not form complexes with suxamethonium and benzylisoquinolinium muscle relaxants (mivacurium, atracurium and cisatracurium), it cannot be used to reverse these agents [67]. Further, factors such as age [68], cardiac output [69], increased stress due to surgery and pregnancy [35, 56] may contribute to delayed return of muscle power following sugammadex administration.^7^. In fact, even in routine surgical population, in spite of reversing with sugammadex, 2% of the patients were found to have residual paralysis (TOF < 0.9) in the recovery room [70]^7^. Fluctuations in muscle power may occur even after seemingly adequate reversal with sugammadex due to the redistribution of unbound muscle relaxant from the peripheral to the central compartment causing a rebound of blockade [71]. Despite the rapid reversal, there is no firm evidence to prove superiority of sugammadex over neostigmine in the prevention of postoperative pulmonary complications according to a recent review [72].

There have been reports of suspected hypersensitivity reactions to sugammadex [73, 74] but more evidence is needed in this regard to confirm its true incidence. In addition, there are concerns about displacement and capturing interactions with sugammadex. In particular, sugammadex may capture the prostagenic compound in oral contraceptive making it less effective [10]. Sugammadex is not also recommended for patients with severe renal impairment or those on dialysis [75] although evidence suggests that the complex with rocuronium can be removed by haemodialysis [76].

### Limitations of the review

There are several limitations to this review. As this review summarizes the findings of various case reports, there are inherent drawbacks such as missing information, inability to draw inferences on causality and publication bias [77]. Non-English reports, abstracts without full texts and pediatric case reports are not included in this review. Since the primary goal of this article is to investigate the use of sugammadex patients in neuromuscular disorders and its clinical considerations, details on the disease severity and medications in every reported case were avoided. Since based on case reports, it has not been possible to provide conclusive evidence on the correct dose and timing of administration of sugammadex in patients with neuromuscular disorders.

## Conclusion

Anesthetic management of patients with neuromuscular disorders is challenging due to the variability in the type, severity of the disorder and the extent of dysfunction in various muscle groups and their sensitivity to muscle relaxants in each patient.

Multiple case reports have been published describing the successful reversal of rocuronium with sugammadex in patients with neuromuscular disorders, however, there are also reports of adverse reactions and instances of inadequate reversal with administration of sugammadex. Currently, as there is limited knowledge on optimal dosing and timing of administration of sugammadex, a similar unpredictability in response also seem to occur with the use of sugammadex in this cohort of patients. Hence despite the advantages of sugammadex in this high-risk group of patients, it is strongly recommended to use quantitative neuromuscular monitoring to ensure complete recovery from the effects of steroidal muscle relaxants and to exercise extended postoperative supervision in these patients.

## Data Availability

The datasets used and analyzed during the current study are available in the text.

## Abbreviations

ALS: Amyotrophic lateral sclerosis
APM: Adductor pollicis
CSM: Corrugator supercilii
MeSH: Medical Subject Headings
PM: Progressive muscular atrophy
PRISMA: Preferred reporting items for systematic reviews and meta-analyses
T1: First twitch

## Footnotes

1 Patients with neuromuscular disorders have a high risk of postoperative respiratory complications including respiratory failure.

2 Sugammadex should be strongly considered as a safer and effective alternative to neostigmine in the reversal of steroidal muscle relaxants.

3 Reduced dose of rocuronium has been used in these patients while the standard recommended dose of sugammadex has been successfully used to reverse muscle relaxants in the majority of the published case reports.

4 Variations from normal recovery patterns of muscle strength following administration of reversal agent have been observed in patients with neuromuscular diseases. In myasthenia gravis especially in ocular myasthenia, both spontaneously and with administration of sugammadex, earlier recovery of TOF ratio versus first twitch height and earlier recovery at corrugator supercilii muscle versus adductor pollicis muscle were observed. Hence, monitoring TOF ratio as well as the recovery of first twitch height to baseline at both the muscles is recommended.

5 Discordance between TOF ratios and clinical recovery have been reported in amyotrophic lateral sclerosis, which has been stated to be related to severity of disease and type of muscles involved.

6 Quantitative neuromuscular monitor is essential in the management of these patients with a TOF ratio of > 0.9 at peripheral muscles before extubation, to ensure adequate pharyngeal function and airway protection as well as to prevent complications such as atelectasis and pneumonia.

7 Since delayed or failed recurarisation and long recovery times have been reported with adequately dosed sugammadex reversal even in normal surgical patients, presence of additional factors such as renal dysfunction, temperature fluctuation, acid-base or electrolyte imbalances in these patients or interaction with other medications (i.e. magnesium, baclofen,) necessitate prolonged post-operative observation, especially for respiratory insufficiency in patients with neuromuscular diseases.
